# Extracorporeal membrane oxygenation in acute respiratory distress syndrome caused by elderly tuberculous meningitis: a case report and review of the literature

**DOI:** 10.3389/fmed.2024.1457413

**Published:** 2024-09-18

**Authors:** Zhaokun Shi, Xue Zhu, Wenwei Gao, Shuhui Yu, Liying Zhan

**Affiliations:** Department of Critical Care Medicine, Renmin Hospital of Wuhan University, Wuhan, China

**Keywords:** tuberculous, meningitis, ARDS, extracorporeal membrane oxygenation, anti-tuberculosis therapy

## Abstract

Diagnosing and clinical management of tuberculous meningitis (TBM) are still challenging for clinicians. Analysis of cerebrospinal fluid (CSF) is an important diagnostic tool for patients with suspected central nervous system (CNS) diseases. Acute respiratory distress syndrome (ARDS), an inflammatory lung injury, can be treated by mechanical ventilation, fluid management, and even extracorporeal membrane oxygenation (ECMO). In addition, metagenomic next-generation sequencing (mNGS) can facilitate the detection of atypical, rare pathogens in clinical specimens. We report a case of a 65-year-old man with ARDS caused by TBM. He was admitted with a fever and shaking. Despite aggressive initial treatment, the patient progressed rapidly and developed ARDS. Without positive results of mNGS and culture, anti-tuberculosis (TB) treatment was started. In order to improve oxygenation levels, he was placed on veno-venous ECMO for 8 days. On day 47, the tracheotomy catheter was pulled out and sealed. The patient was conscious and could communicate with family members as normal.

## 1 Introduction

Tuberculosis (TB) is an infectious disease that is caused by bacteria (*Mycobacterium tuberculosis*). The most serious form of TB is tuberculous meningitis (TBM), which has a high mortality rate. According to World Health Organization statistics, there were 1.3 million people died of TB in 2022 (1). It is reported that TBM accounts for approximately 1% of all TB cases globally (2). Due to atypical symptoms and lack of etiological evidence, the diagnosis of TBM is extremely difficult. In addition, acute respiratory distress syndrome (ARDS) caused by TBM has never been reported. ARDS, characterized by severe dyspnea, acute hypoxemia, and bilateral pulmonary edema (3), is often caused by trauma, infection, and other non-cardiac causes. The crude incidence of ARDS in the United States is 64.2–78.9 cases per 100,000 person-years, and the overall mortality rate is 43% (4–7). Mechanical ventilation, prone ventilation, and fluid management are common treatments for ARDS. In recent years, veno-venous extracorporeal membrane oxygenation (V-V ECMO) has been proven to be successfully used in the treatment of ARDS (7). For the patient in the current study, ECMO was beneficial to the recovery of lung injury. ECMO is an effective technique to improve arterial oxygenation, although the patient eventually died of myocardial infarction. This is the first reported case of ARDS caused by TBM and successfully treated by ECMO.

## 2 Case presentation

**Figure 1:**
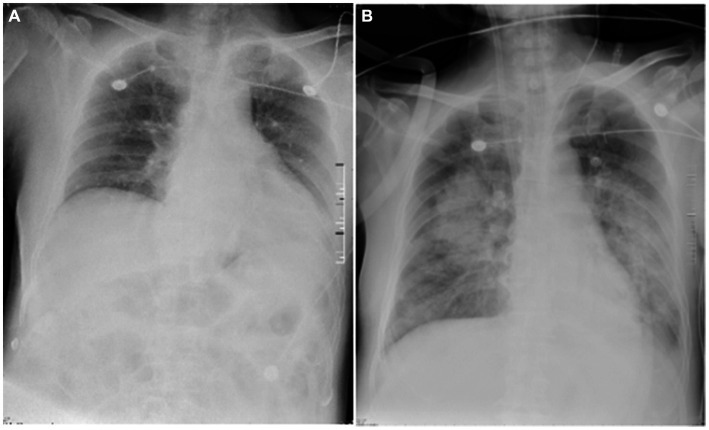
Chest X-ray images of the patient. The chest X-ray image **(A)**, obtained on day 1, showed enhanced lung markings in both lungs and an enlarged heart shadow. No lesions were found in the lungs. The diaphragmatic surface of each lung was smooth, and costophrenic angles were in shape. The chest X-ray image **(B)**, obtained on day 7, showed multiple patchy and flaky shadows in the lungs. The diaphragmatic surface and costodiaphragmatic angle of the left lung were blurred. The diaphragmatic surface and costophrenic angle in the right were smooth.

A 68-year-old retired lawyer was admitted to the ICU with a history of high blood pressure and coronary heart disease treated with percutaneous coronary intervention (PCI), and he had been suffering from intermittent body shaking, fever, headache, neck stiffness, and generalized myalgia for 3 days. The whole body shaking occurred after the completion of thermal annealing. On the day of admission, the patient was conscious and could speak with slow speech. His body temperature was 38.3°C, blood pressure was 167/81 mmHg, pulse was 71 beats per minute, respiratory rate was 20 breaths per minute, and oxygen saturation was 100%, and he was treated with a nasal cannula. Auscultation of heart and lung sounds was normal, with no murmurs or crackles. The muscle score was Grade 3. Except for neck stiffness, the other pathologic reflexes (e.g., Babinski and Oppenheim) were negative. Blood tests revealed a white blood cell count of 8.639 × 10^9^/L [reference value: (3.5–9.5) × 10^9^/L], neutrophil count of 7.06 × 10^9^/L [(1.8–6.3) × 109/L], lymphocyte count of 0.076 × 10^9^/L [(1.1–3.2) × 10^9^/L], hemoglobin of 133 g/L, C-reactive protein of <3 mg/L, serum amyloid A protein (SAA) of <5 mg/L, and procalcitonin (PCT) of 0.044 ng/mL. Galactomannan enzyme immunoassay (GM-EIA) was 0.038 and fungus (1-3)-β-D-glucan test was 66.538 pg/mL. Cytomegalovirus (CMV) and Epstein–Barr virus (EBV) DNA were all negative. S-100 protein, a brain-specific protein in the central nervous system (CNS), was 228 pg/mL. Neuron-specific enolase (NSE), a glycolytic enzyme released upon neuronal damage, was 26.6 ng/mL. Interleukin-6 (IL-6) values were increased by 160.69 pg/mL, and the concentration of interleukin-10 (IL-10) was 29.95 pg/mL. Blood culture and sputum culture were also performed. The chest X-ray of the patient showed no abnormality on the lung (Figure 1A). In addition, lumbar puncture and cerebrospinal fluid (CSF) analysis were used to assist in determining the etiology of the neurological deficit. The CSF was a yellowish and slightly cloudy fluid with a protein concentration of 2,150 mg/L (120–600 mg/L) and a normal glucose level (Table 1). No abnormality was found in acid-fast staining or ink staining. Epileptic waves were observed on the electroencephalogram (EEG). Empirical intravenous antibiotics and antiepileptic therapy were started.

**Table 1:** Changes of cerebrospinal fluid.

	D2	D4	D16	D18	D21	D22	D33
White blood cells (×10^6^/L)	37	70	59	0	1	0	12
Protein (mg/L)	2,150	2054.1	760	1106.9	640	630	610
Glucose (mmol/L)	3.46	-	3.03	4.37	3.51	3.78	4.60
Chloride (mmol/L)	112	118.5	121	127.1	119	118	114

**Figure 2:**
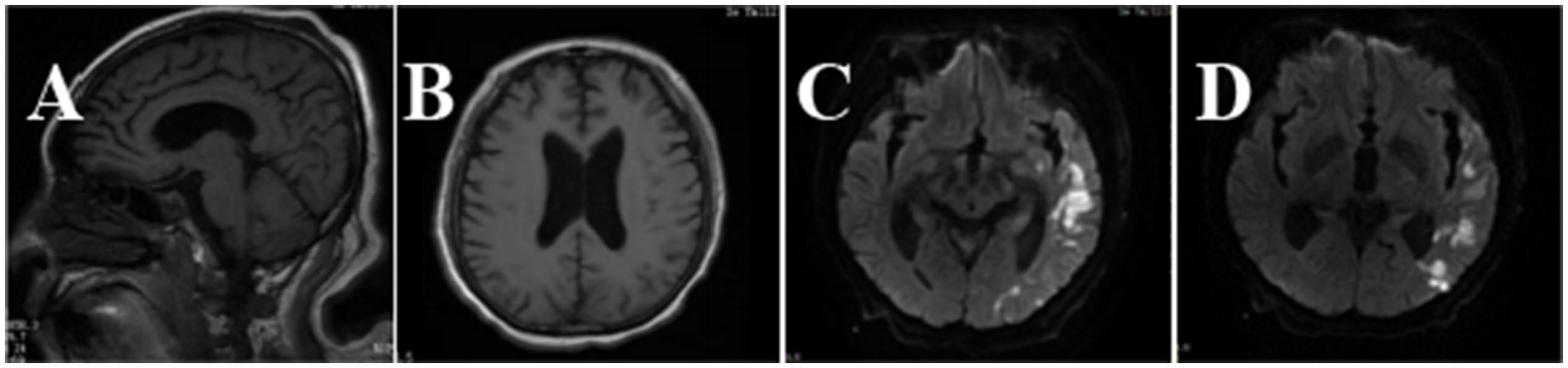
Brain MRI images of the patient. In the T1-weighted image **(A)**, there was an adhesion around the mesencephalic aqueduct. In the T2-weighted image **(B)**, the ventricular system was not enlarged. Brain sulci and brain cleft were widened. In diffusion-weighted imaging (DWI) **(C,D)**, hyperintense signals could be seen in the left temporo-parieto-occipital lobe, bilateral frontal lobe, and corona radiata.

Fever and shaking recurred on day 3. The oxygen saturation level of this patient ranged from 70 to 90%, and an arterial blood gas (ABG) measurement revealed a partial pressure of oxygen (PO_2_) of 52 mmHg. On day 4, endotracheal intubation and mechanical ventilation were performed on the patient with hypoxemic respiratory failure and decreased level of consciousness, with positive end-expiratory pressure (PEEP) of 10 mmHg and a fraction of inspiration oxygen (FiO_2_) of 100%. Unlike the previous chest radiograph, pulmonary edema was graded depending on the chest X-ray, clearly abnormal (Figure 1B). CSF from the second lumbar puncture turned into a clear and light-yellow fluid, and it was still an elevated protein level in the CSF (Table 1). Based on the current condition of the patient, tuberculous meningitis (TBM) was highly suspected. Antiviral and anti-tuberculosis drugs were also administered. However, the range of oxygen saturation level was between 75 and 90%, the heart rate was between 130 and 150 bpm, and blood pressure was maintained by high doses of vasopressor therapy (2.4 mg/h of norepinephrine and 16 mg/h of aramine). On day 5, veno-venous extracorporeal membrane oxygenation (V-V ECMO) was commenced, providing support for the lungs with a blood flow of 3.2 L/min and 100% O_2_. The width of the venous cannula used in ECMO for diverting blood flow was 1.1 cm. The magnetic resonance imaging (MRI) of the brain showed high signal intensity in the right basal ganglia, radiating crown area, and the left corpus callosum area. Some dot-line-enhanced signals were detected in the cerebral sulci. There was an adhesion in the mesencephalic aqueduct. The ventricular system of the brain did not become enlarged at the initial stage (Figure 2).

**Figure 3:**
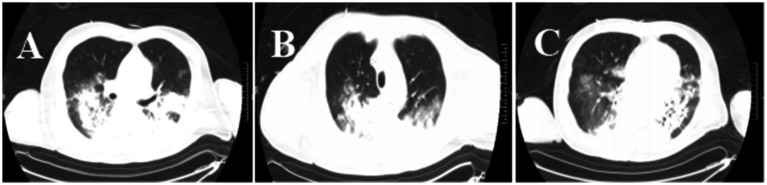
Chest CT images of the patient. The CT chest images **(A–C)** showed that the texture of both lungs was enhanced, and patchy shadows, increased density shadows, and ground-glass shadows in the lungs were seen. There was partial consolidation in the lung tissue.

**Figure 4:**
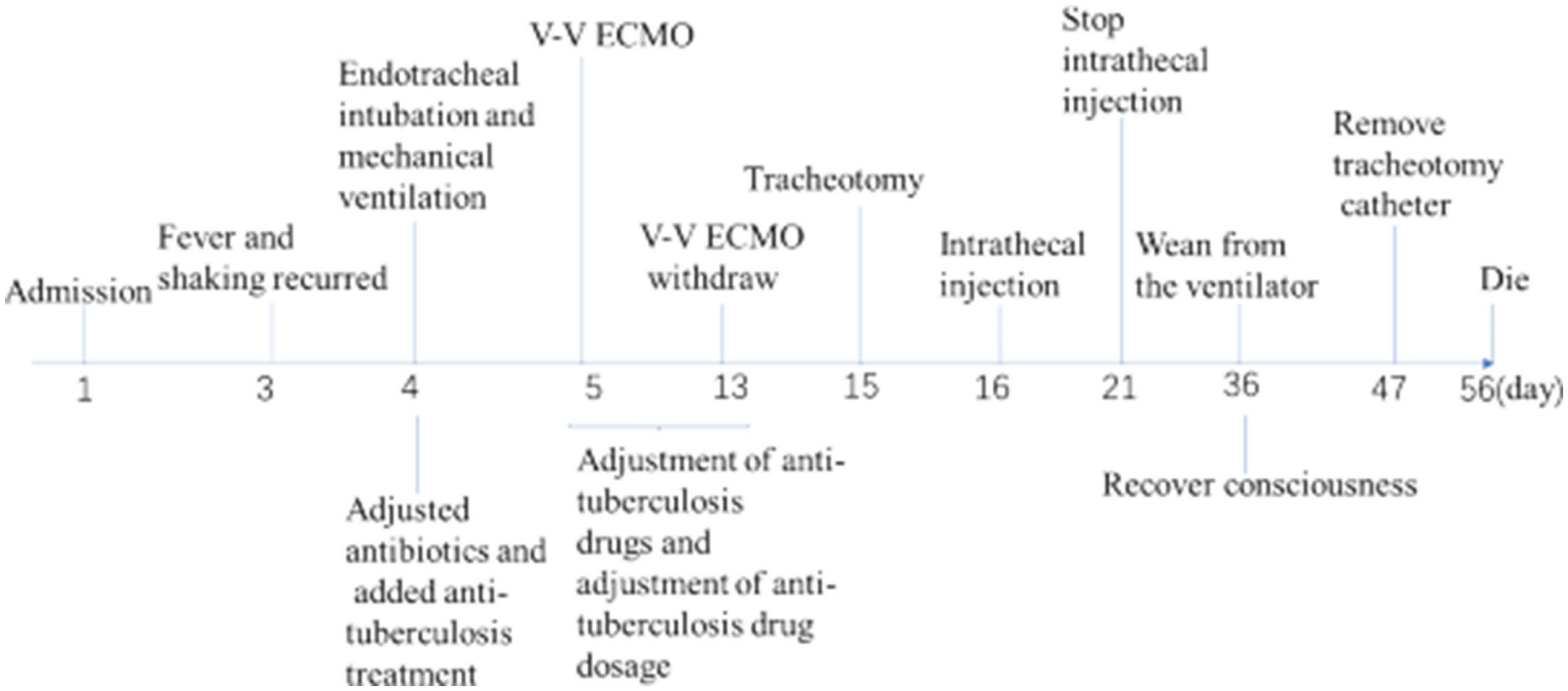
The timeline of the patient’s disease progression.

Moreover, increased lung markings were observed on the computed tomography (CT) scan imaging (Figure 3). On day 6, the lactate level in the blood was above 15 mmol/L. After rehydration, dialysis, and other treatments, the heart rate of the patient dropped to 90–110 bpm. The blood flow of ECMO was slowly increased to approximately 3.5 L/min, and the dose of vasoactive drugs was significantly reduced. On day 7, the blood lactate dropped to 3.2 mmol/L, and the oxygenation index was 118 mmHg. For further evaluation of the lungs and heart, a chest X-ray was performed again. The chest radiography indicated pulmonary edema and suggested an infection in the lungs of this patient (Figure 4B). Under bronchoscopy, large amounts of white mucus could be seen in the main airway. However, pulmonary auscultation did not detect any dry or wet rales. The oxygenation index gradually increased to 225 mmHg after the use of the prone position in this patient. On day 13, ECMO was withdrawn. On day 15, the patient was still difficult to wean from invasive mechanical ventilation, so the tracheotomy was performed (Figure 4).

The Oxford University Clinical Research Unit found that adjunctive treatment with intrathecal dexamethasone improves survival in TBM patients over 14 years of age (8). In addition, adjunct adjunctive isoniazid with dexamethasone anti-TB can enhance therapeutic outcomes and reduce adverse reaction rates in adult TBM patients (9). On day 16, intrathecal injections of isoniazid and dexamethasone were given intermittently in the ICU. On day 21, the protein level of the CSF decreased greatly, and the patient was able to open his eyes to a painful stimulus after the withdrawal of sedatives. The next day, the intrathecal catheter was removed. On day 25, vasoactive medications were reduced and eventually discontinued. On day 36, the patient recovered consciousness and was completely weaned from the ventilator. On day 47, the tracheotomy catheter was sealed and removed. Unfortunately, on day 56, the patient had a sudden onset of irritability, delirium, and yelling, and an electrocardiogram showed acute myocardial infarction. Based on the patient’s history of coronary heart disease, it was considered that the patient’s death was not directly related to the treatment course. Finally, the patient had died. The final diagnosis of the patient’s death was acute myocardial infarction with ARDS due to TBM.

## 3 Discussion and conclusion

Tuberculous meningitis is a critical disease that is difficult to diagnose clinically. Started with atypical symptoms, it is difficult to distinguish from other tuberculosis of the nervous system (10), such as tuberculoma and tuberculous brain abscess, especially for elderly patients (11). The survival of elderly TBM patients was found dismal, and there are significant differences in clinical, CSF, and radiological characteristics between the elderly and young (12). In general, the infection rate of tuberculous meningitis is higher in children under 2 years of age and less in adults, and all have a high risk of death (13, 14). Some patients with tuberculous meningitis had cognitive impairment (15). A Chinese study on the differential diagnosis of tuberculous meningitis and bacterial meningitis found that the proportion of lymphocytes in the CSF was significantly higher in the TBM group than in the bacterial meningitis (BM) group, while the CSF chlorine, nucleated cell count, and the proportion of neutrophils were significantly low in the TBM group (16). This patient did not have the typical changes in the lymphocyte, nucleated cell, neutrophil count, and chloride concentration in the cerebrospinal fluid. However, his anti-tuberculosis treatment was very effective.

Metagenomic next-generation sequencing has significant advantages for the detection of pulmonary infections, especially for mixed microbial infections and rare microbial infections (17). This technology can amplify and sequence nucleic acids (DNA or RNA) of atypical pathogens quickly and accurately, providing detailed characterization of genomes. mNGS analysis is helpful for the treatment of pulmonary infections in critically ill patients. It had been reported that metagenomic next-generation sequencing (mNGS) had high specificity in the diagnosis of tuberculous meningitis (18). In this case, we performed mNGS detection in the sputum, bronchoalveolar lavage fluid, CSF, and blood samples many times, but the results were all negative. Although the patient had negative mNGS results, typical changes in TBM in the MRI brain had been found. Therefore, on the basis of imaging, mNGS findings, laboratory results (including CSF examinations), and clinical manifestations, the TBM diagnosis should be considered.

Tuberculous meningitis requires regular anti-tuberculous therapy for 6 months (19). Based on the possible presence of drug-resistant TBM, a combination of levofloxacin, kanamycin, ethionamide, linezolid, and pyrazinamide would be an appropriate regimen because of the great CSF profile of most of these drugs (20, 21). The mortality of TBM patients was high and was associated with delay in initiation of treatment, older age, HIV infection, and higher disease severity at admission (22). Treated with antituberculosis medications and other management, the level of consciousness in this patient was improved gradually and he could communicate with family members normally. Considering anti-tuberculosis drug-induced hepatotoxicity and nephrotoxicity, the lowest doses of isoniazid, rifampin, and pyrazinamide were used. However, on day 7, the patient suffered from acute liver injury and kidney injury. In the subsequent treatment, medications that protect the kidneys and liver were added, and the liver and kidney function returned to normal gradually.

A study showed that intrathecally administered isoniazid and hormones could reduce the mortality of TBM in adults (23). Dexamethasone treatment reduces mortality in patients with tuberculous meningitis, reduces the exudation of inflammation, decreases the levels of cytokines in brain tissue, and reduces the likelihood of complications (24, 25). Thus, intrathecal isoniazid and dexamethasone administration were added. Moreover, studies assessing the pharmacokinetics of anti-tuberculosis drugs found that low doses of these drugs were difficult to reach the target CSF concentration (26–29). Thus, anti-tuberculosis drugs were adjusted to rifampicin, isoniazid, pyrazinamide, streptomycin, and amikacin, and the oral doses taken by the patient were increased. After multifaceted interventions, the CSF gradually became clear, and the concentration of protein in the CSF decreased to 610 mg/L.

At the time of admission, the patient was conscious and had no dyspnea. However, in the process of diagnosis and treatment, ARDS was developed gradually. We speculated that the autonomic nervous system played an important role in the development of ARDS in this case. The imbalance between ventilation (V) and perfusion (Q) and the release of massive catecholamines, occurring after the activation of the sympathetic nervous system (30), lead to hypoxemia and neurogenic pulmonary edema. Additionally, dysregulated inflammation and over-expression of inflammatory factors were related to the injury of the pulmonary vascular endothelium and alveolar epithelium in patients with TBM, finally resulting in respiratory failure (31). Mechanical ventilation, prone ventilation, and fluid management are common treatments for ARDS. In recent years, veno-venous extracorporeal membrane oxygenation (V-V ECMO) has been proven to reduce the incidence of ventilator-induced lung injury (V-ILI) in severe ARDS patients with preserved cardiac function (7). ECMO is a viable treatment option for patients with TB. A German study found that TB patients were treated with ECMO mainly because of respiratory failure or heart failure, including 83.7% of TB patients who received V-V ECMO and 24.3% of TB patients who received V-A ECMO (32). V-V ECMO has been used in the literature on respiratory failure or ARDS caused by tuberculosis pneumonia, which accounts for the majority of tuberculosis infections (33–36). VAV ECMO has also been used for ARDS and septic myocarditis due to tuberculosis (37). However, we have not seen relevant studies and literature reports on the use of ECMO in the treatment of TBM. ECMO pumps blood out of the body and sends it to a heart-lung machine, removing carbon dioxide and adding oxygen, and then pumps the blood back to the body. In this case, on day 5, V-V ECMO was commenced in the ICU, with an initial flow rate of 3.2 L/min. During the ECMO, the bronchoscopy revealed a large amount of white sputum in the airway. Then, intermittent prone positioning was performed to improve the gas exchange in the lungs. However, it has been reported in the literature that prone ventilation during ECMO did not decrease the time of ECMO weaning (38). After adjustment and evaluation, ECMO was withdrawn on day 13. Whether the start time of V-V ECMO could be advanced and the duration of V-V ECMO could be shortened still needs further study and research.

In conclusion, these findings ultimately lead us to the correct diagnosis of TBM. This is the first reported case of ARDS caused by TBM and successfully treated by ECMO. Although the patient eventually died of myocardial infarction, V-V ECMO is a valuable adjuvant therapy. When clinicians encounter similar cases, CSF analysis and mNGS results are essential and can be repeated if necessary for early diagnosis and treatment. Furthermore, it is necessary to adjust the treatment and drug dosage according to the results of the laboratory examination as early as possible. As a life support technology, veno-venous (V-V) or venous–arterial (V-A) ECMO is recommended for patients with respiratory failure or circulatory failure.

## Data Availability

The original contributions presented in the study are included in the article/supplementary material, further inquiries can be directed to the corresponding author.
